# Activation of BK Channel Contributes to PL-Induced Mesenchymal Stem Cell Migration

**DOI:** 10.3389/fphys.2020.00210

**Published:** 2020-03-24

**Authors:** Santiago Echeverry, Adriana Grismaldo, Charles Sánchez, Cristian Sierra, Juan C. Henao, Sara T. Granados, Jhon-Jairo Sutachán, Yolima P. Torres

**Affiliations:** Departamento de Nutrición y Bioquímica, Facultad de Ciencias, Pontificia Universidad Javeriana, Bogotá, Colombia

**Keywords:** BK channel, platelet lysate, cell migration, ion channels, mesenchymal stem cells

## Abstract

Due to their capacity to proliferate, migrate, and differentiate, mesenchymal stem cells (MSCs) are considered to be good candidates for regenerative medicine applications. The mechanisms underlying proliferation and differentiation of MSCs have been studied. However, much less is known about the mechanisms regulating the migration of MSCs. Platelet lysate (PL), a supplement used to promote cell expansion, has been shown to promote MSCs migration; however, the underlying mechanism are unknown. Here, by using adipose-derived rat MSCs (rMSCs) and the scratch assay in the absence and presence of various BK channels modulators, we evaluated the role of BK channels in mediating the PL-stimulated migration of rMSCs. We found that 5% PL increased rMSCs migration, and this effect was blocked by the addition of the BK channel selective antagonist Iberiotoxin (IBTX). In the absence of PL, the BK channel agonist NS1619, stimulated rMSCs migration to similar level as 5% PL. Addition of both NS1619 and 5% PL resulted in an increase in rMSCs migration, that was higher than when either one was added individually. From whole-cell recordings, it was found that the addition of 5% PL increased the magnitude of BK current density. By using Western blot and flow cytometry, it was found that PL did not affect the expression of BK channels. Together, our results indicate that as shown in other cell types, activation of BK channels by themselves also promote rMSC migration, and show that activation of BK channels contribute to the observed PL-induced increase in migration of rMSC.

## Introduction

The efficient migration of stem cells and their subsequent differentiation and long-term survival are essential characteristics for a successful use of stem cell-mediated treatments (Müller et al., 2008; Carney and Shah, 2011; Serakinci et al., 2014). Mesenchymal stem cells (MSCs) are considered to be good candidates for regenerative medicine because they show these properties. Very little information is available regarding the mechanisms underlying MSCS migration. In other cell systems, it has been found that cell migration is controlled by a complex signal network that consist of chemical, electrical, and mechanical regulators (Li and Jiang, 2011; Herzmann et al., 2016). Platelet lysates (PL) contain bioactive constituents such as growth factors, chemoattractants, and mitogens that modulate migration of different cell types, including MSCs (Barsotti et al., 2013). In MSCs, PL induces the production of paracrine factors that promote bone regeneration and also stimulates cell migration (Chevallier et al., 2010; Leotot et al., 2013; Ulivi et al., 2014). However, the mechanisms underlying the PL-induced increase in migration of MSCs remain unclear. A better understanding of the mechanisms underlying migration of MSCs will help to support their use in conditions requiring cell migration, such as in tissue repair.

Ion channels are involved in cell migration, as well in proliferation and differentiation of various cell types, including airway smooth muscle cells, endothelia cells, and glioblastoma (Rosa et al., 2016; Tajhya et al., 2016). However, the contribution of a given channel on these properties depends on the cell type. The transient receptor potential (TRP) channels TRPM8 and TRPA1 are involved in the platelet-derived growth factor (PDGF)-induced proliferation and migration of airway smooth muscle cells by a mechanism that involves downregulation of their expression (Zhang et al., 2016). In contrast, increased BK and TRPM8 channel activity has been associated with a rise in human glioblastoma cells migration by regulating intracellular Ca^2+^ concentration levels (Wondergem and Bartley, 2009). Activation of large conductance voltage-and-Ca^2 +^ -activated K^+^ channels (BK) channels, through intracellular signaling pathways, have been identified as an essential step in the basic fibroblast growth factor (bFGF)-induced proliferation of endothelial cells (Kuhlmann et al., 2004; Steinle et al., 2011). While, the BK channel blocker paxilline promoted an increase in the proliferation of human pluripotent stem cell-derived mesenchymal stromal cells (Zhao et al., 2013).

Mesenchymal stem cells express a broad range of ion channels, including BK channels (Heubach et al., 2004). This channel is ubiquitously expressed and activated by different stimulus including changes in cytoplasmic Ca^2+^ concentration, membrane depolarization and phosphorylation. BK channels are α subunit tetramers that can be associated with β subunits (β1-4), which modify their biophysical and pharmacological properties (Latorre et al., 2017). However, to our knowledge there are no reports investigating the role of BK channels in rMSC migration. In this study, we investigated if BK channels were involved in migration of rMSCs and the possible role of these channels in the effect of PL-induced increase in rMSC migration. The results of this study will contribute to the understanding of MSC migration, allowing for their better manipulation when used in regenerative medicine.

## Materials and Methods

### Reagents

The BK channel antagonist, Iberiotoxin (IBTX), and agonist, NS1619 (Alomone Labs, Jerusalem, Israel), were solubilized in water and dimethyl sulfoxide, respectively. CD45-allophycocyanin (APC), CD90-phycoerythrin (PE), and CD29-Alexa Fluor 488 conjugated antibodies were purchased from Biolegend, San Diego, CA, United States. Anti-sloβ1 (NB300-535) and anti-K_Ca_1.1 (NBP1-46701), directed against β1 and α subunits, respectively, were from Alomone Labs and Novus biological. Anti-rabbit Alexa Fluor 488 was purchased from Invitrogen, Carlsbad, CA, United States.

### Cell Isolation

Rat MSCs were obtained from abdominal fat tissue of 2–3 months old pregnant Wistar rats used in other experiments, to minimize animal use. All experiments were approved by the Ethics Committee at the Pontificia Universidad Javeriana, Bogotá, Colombia, and conducted according to the institutional guidelines for the use of experimental animals. Adipose tissue was washed with phosphate-buffered saline (PBS) supplemented with penicillin/streptomycin (P/S) 5% and treated with 0.075% collagenase type I in PBS plus 2% P/S at 37°C. After 1 h, collagenase was neutralized by adding Dulbecco’s Modified Eagle’s Medium (DMEM) supplemented with 10% fetal bovine serum (FBS) and P/S 2%. The sample was pipetted up and down several times to fragment the tissue aggregates and then filtered and centrifuged at 360 × *g* for 5 min. The sample was then resuspended in DMEM supplemented with 10% FBS and maintained at 37°C and 5% CO_2_. After 72–96 h, the cells were washed twice with PBS to remove any non-MSCs. For all the assays, MSCs from passages 2–7 were used assays (Bunnell et al., 2008).

### rMSC Characterization

Rat MSCs were characterized by evaluating the expression of the surface markers CD45, CD29, and CD90 by flow cytometry (Guava easyCyte; Millipore, Billerica, MA, United States). Cells were trypsinized after reaching 90% confluence, centrifuged at 360 × *g* for 5 min and suspended in DMEM at a density of 2 × 10^5^ cells/ml. Cells were fixed with 4% formaldehyde and centrifuged at 360 × *g* for 5 min. The pellet was suspended in methanol; left overnight at −20°C, washed in PBS plus 2% FBS, and incubated with antibodies CD45-APC, CD90-PE, and CD29-Alexa Fluor 488 for 1 h. Samples were evaluated using a Guava cytometer (Guava easyCyte; Millipore) and analyzed with the Guava Incyte Software.

### PL Preparation

Platelets were isolated from healthy volunteers after obtaining the written informed consent and with the approval of the Institutional Ethics Committee at the Pontificia Universidad Javeriana. Blood was collected and centrifuged at 800 × *g* for 8 min. 50% of the plasma volume was removed to produce a platelet-rich plasma, which was then centrifuged, the plasma was removed, and the platelet pellet was resuspended in PBS. Finally, the platelets were frozen to −80°C and thawed to 37°C (this cycle was repeat for three times), and then used in subsequent experiments.

### BK Channel Expression

Total expression of BK subunits was evaluated by flow cytometry and Western blot, using specific antibodies in permeabilized cells. For flow cytometry, cells at 80–90% confluence were detached, centrifuged at 360 × *g* for 5 min, suspended in DMEM at a density of 2 × 10^5^ cells/ml, fixed with 4% formaldehyde, and centrifuged again at 360 × *g* for 5 min. Then, cell pellet was resuspended in methanol and left overnight at −20°C. Cells were washed in PBS–2% FBS, centrifuged at 360 × *g* for 5 min, and incubated with primary antibody for 1 h (anti-K_Ca_1.1; anti-sloβ1), followed by secondary antibody (anti-rabbit Alexa Fluor 488; Invitrogen) for 30 min, and then washed in PBS buffer. Cells incubated only with secondary antibody were used as a control. Samples were evaluated using a Guava cytometer (Guava easyCyte; Millipore) and analyzed with Guava Incyte software. All experiments were performed in triplicate, and the number of samples (*n*) was 5.

For Western blot assays, MSCs were trypsinized, washed twice with PBS and lysed at 4°C with RIPA buffer containing protease/phosphatase inhibitor cocktail. Lysates were centrifugated (16000 rpm for 1 min at 4°C), and protein concentration was measured by using the Bicinchoninic acid kit (ThermoFisher Scientific). 20 μg of protein were separated by 10% sodium dodecyl sulfate-polyacrylamide gel electrophoresis, transferred to a PVDF membrane (BioRad) using standard techniques. The membranes were blocked with Tris-buffered saline with Tween (TBS-T) 0.1% + 5% milk, and stained with anti-K_Ca_1.1; anti-sloβ1 antibody prepared in blocking buffer with 5% Bovine serum albumin (1:500). The membrane was then washed three times with TBS-T 0.1%, incubated for 1 h with horseradish peroxidase-conjugated secondary antibody (1:6000; Goat anti-rabbit IgG, Invitrogen) and washed with TBS-T 0.1% for 5 min. Blots were visualized by using a chemiluminescence substrate (SuperSignal West Dura Extended Duration Substrate).

In order to evaluate BK channel surface expression, we used flow cytometry in non-permeabilized cells. Cells at 80%–90% confluence were detached, centrifuged at 360 × *g* for 5 min and suspended in DMEM at a density of 2 × 10^5^ cells/ml. Cells were fixed with 4% formaldehyde, washed in PBS–2% FBS, centrifuged at 360 × *g* for 5 min, incubated with primary antibody for 1 h (anti-K_Ca_1.1; anti-sloβ1), followed by secondary antibody (anti-rabbit Alexa Fluor 488; Invitrogen) for 30 min, and then washed in PBS buffer. Cells incubated only with secondary antibody were used as a control. Samples were evaluated using a Guava cytometer (Guava easyCyte; Millipore) and analyzed with Guava Incyte software. All experiments were performed in triplicate, and the number of samples (*n*) was 5.

### Cell Viability Assay

Rat MSCs were detached and plated in 12-well plates at a density of 2 × 10^5^ cells/well. The cells were then subjected to the following treatments after 16 h: IBTX 10 nM; NS1619 10 μM; 5% PL plus heparin (1% v/v); IBTX 10 nM + 5% PL plus heparin (1% v/v); and NS1619 10 μM + 5% PL plus heparin (1% v/v). Cells treated with doxorubicin 30 μM were used as a positive control and cells without treatment were used as a negative control. After 24 h, the cells were detached, stained with propidium iodide, and analyzed by flow cytometry. All experiments were performed in triplicate and *n* was 5.

### Migration Assay

Rat MSCs were plated in 24-well plates in DMEM. When the cells reached confluence, a linear wound was made by scratching the cell monolayer with a sterile 1000 μl plastic pipette tip. Cells were incubated with fresh medium for 24 h in the presence of BK channel modulators (IBTX 10 nM and NS1619 3 and 10 μM), with or without 5% PL (v/v) plus heparin (1% v/v). Cells without treatment were used as controls. The area of the wound gap was then observed and photographed under an inverted microscope (20X) (Nikon, Tokyo, Japan) and cells on the wound area were counted at 0 and 24 h after scratching to assess cell mobility under different treatment conditions. Number of cells in wound area after each treatment was dividing by number of cells in control experiments to analyze changes in migration induced by the treatments. Five experiments were performed in triplicate.

### Electrophysiology

Whole-cell currents were recorded using the patch-clamp technique. Cells were detached and seeded onto glass coverslips 30 min before assay. Recordings were carried out at room temperature (20–22°C) in a bath solution containing 143 mM NaCl, 5.4 mM KCl, 1.8 mM CaCl_2_, 0.5 mM MgCl_2_, and 5 mM [4-(2-hydroxyethyl)-1-piperazineethanesulfonic acid] HEPES, pH 7.4. The pipette solution consisted of 150 mM KCl, 1 mM MgCl_2_, 5 mM ethyleneglycol tetraacetic acid, 2 mM Mg-ATP, and 10 mM HEPES, pH 7.4. Pipettes were made from borosilicate glass capillaries (World Precision Instrument, Inc., Sarasota, FL, United States; 1B150F-4), pulled in a horizontal pipette puller (P-97, Sutter Instruments, Novato, CA, United States), and heat polished (MF-830, Narishige, Amityville, NY, United States). The pipette resistance was 3–5 MΩ. Currents were recorded using an EPC-7 amplifier (HEKA, Holliston, MA, United States) and a Nikon TS100 inverted microscope, filtered at 1/5 of the acquisition rate and sampled with an A/D converter (NI-PCIe-6351; National Instruments, Austin, TX, United States). The acquisition software was developed by Dr. Patricio Orio, CINV, Valparaiso, Chile, using the LabView programming environment (National Instruments, Austin, TX, United States). Data analysis was performed using Clampfit 9 software (Molecular Devices, Sunnyvale, CA, United States). Currents were activated by a voltage step protocol with a holding potential of −80 mV, voltage pulses between −100 and +250 mV in 10 mV increments with durations of 70 ms, followed by a step to −80 mV. Assays were carried out after 24 h of treatment with 5% PL and currents were measured in the presence or absence of IBTX 100 nM. Series resistance was compensated, and changes were no greater than 10% during the experiments. To confirm the presence of BK channel current in the recordings, we subtract the current measured in the presence of IBTX from that obtained before the addition of the antagonist.

### Fluorescence Ca^2+^ Imaging

Rat MSCs were loaded with 5 μM Fluo-4AM (Invitrogen) in a solution with 143 mM NaCl, 5.4 mM KCl, 1.8 mM CaCl_2_, 0.5 mM MgCl_2_, and 5 mM HEPES, pH 7.4, for 1 h at 37°C in darkness. Fluorescence measurements were made by using a Zeiss inverted microscope Axio Observer and a AxioCam ICc1 camera. After record basal fluorescence, 5% PL was added by perfusion to the recording chamber. Ionomycin 5 μM was added at the end of the experiment to elicit a maximum Ca^2+^ influx for signal normalization. Changes in fluorescence signal induced by PL were analyzed using ImageJ.

### Statistical Analysis

The results are shown as the mean ± standard error of the mean (SEM). The significance of differences in electrophysiological analysis was determined by analysis of variance with a value of *p* < 0.05 considered significant. *T* test was used to analyze channel expression. ANOVA analysis was used with Kruskal–Wallis *post hoc* to analyze cell viability and migration. *p < 0.05; **p < 0.01; ***p < 0.001; ****p < 0.0001.

## Results

### BK Channels Are Involved in the Effect of PL on rMSCs Migration

**FIGURE 1:**
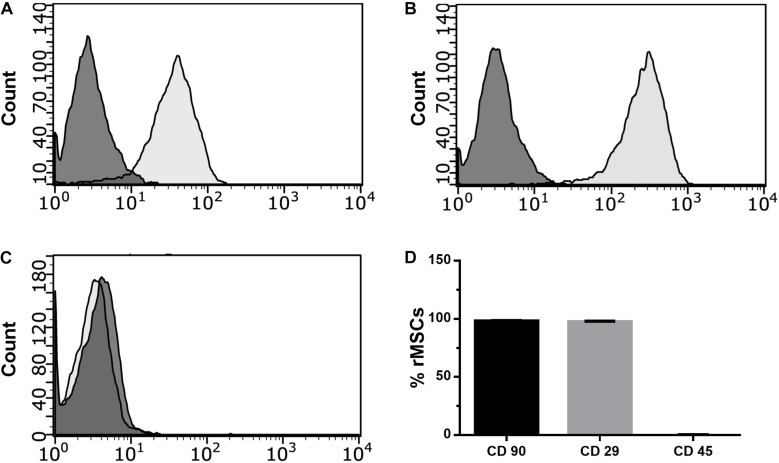
Rat Mesenchymal stem cells immunophenotype characterization. Flow cytometry histograms showing the expression of the antigens **(A)** CD90, **(B)** CD29, and **(C)** CD 45. The figure shows data from one representative experiment. Dark gray: cell without staining; Light gray: Cells with primary antibody. **(D)** Percentage of cells expressing antigens; *n* = 3.

**FIGURE 2:**
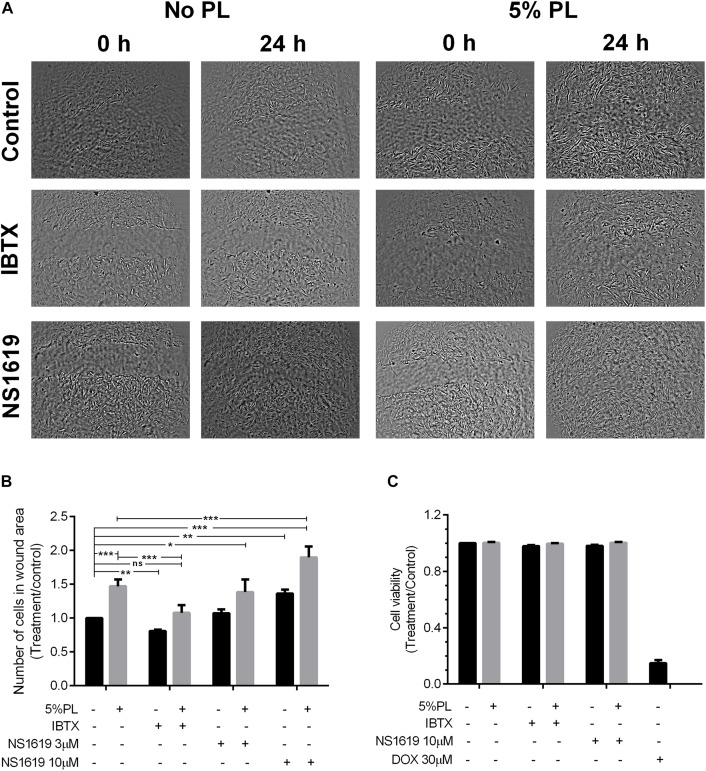
Effect of PL in rMSC migration. **(A)** rMSCs monolayers after scratching at 0 and 24 h in control cells and after treatment with IBTX (10 nM) or NS1619 in presence and absence of 5% PL. **(B)** Number of cells counted in wound area after each treatment was divided by number of cells in wound area in control assay. **(C)** Effect of PL in cell viability after 24 h of treatment with IBTX or NS1619 in the presence and absence of 5% PL. Doxorubicin 30 μM was used as a positive control. Data were normalized to control cells without treatment and shown as mean ± SEM (*n* = 5).

Characterization of rat MSCs (rMSCs) showed that 97–98% of cells were positive for CD90 and CD29, and less than 0.1% were positive for CD45 ruling out a hematopoietic cell contamination and confirming a mesenchymal phenotype of the isolated cells (Figures 1A–D). MSCs exert their function by migrating to places where they are needed in regeneration; thus, maintaining their migratory capacity is an important property of MSCs applications in cell therapy. PL has been shown to have chemoattractant effects on MSCs (Leotot et al., 2013) suggesting that PL could stimulate the migratory properties of these cells. To evaluate whether PL can affect rMSC capacity to migrate, we performed scratch assays on rMSC cultures in the presence and absence of 5% PL (Crespo-diaz et al., 2011). We observed that treatment with 5% PL for 24 h increased rMSCs migration by almost 47% (1 and 1.47 ± 0.04 for control and 5% PL, respectively; *n* = 5, *p* < 0.0001) (Figures 2A,B) suggesting that PL contains factors that stimulate this process. Migration of MSCs is modulated by external cues such as cytokines, chemokines, and growth factors, which regulate several cellular processes (polymerization of actin filaments, cell polarization, and cell membrane protrusion activity) (Paim et al., 2018). Previous studies have shown that PL is rich in growth factors such as PDGF, FGF, and transforming growth factor (TGF) (Mazzucco et al., 2010; Hayon et al., 2013) that can modulate migration by mechanisms involving ion channel activation. For instance, bFGF can increase the open probability of BK channels – an event that has been correlated with the migration of glioblastoma cells (Kuhlmann et al., 2004). To evaluate whether BK channels were involved in the PL-induced migration of rMSCs, cells were treated with the BK channel antagonist IBTX in the presence or absence of PL. IBTX treatment affected rMSC migration in the absence of PL by inducing a decrease in cell migration (1 and 0.81 ± 0.018 for control and IBTX, respectively; *n* = 5; *p* < 0.001; Figures 2A,B) suggesting that BK channels regulate rMSCs migration in basal conditions. We further evaluated the effect that IBTX may have on the migration of cells treated with PL. We found that 10 nM of the BK channel antagonist IBXT abolished the PL-induced migration effect on MSCs (1.47 ± 0.04 and 1.08 ± 0.05 for 5% PL and 5% PL + IBTX, respectively; *n* = 5, *p* < 0.001). Cells treated with 5% PL plus IBTX migrated at a similar level to control cells without PL (1 and 1.08 ± 0.05 for control and 5% PL + IBTX, respectively, *p* > 0.05; Figures 2A,B).

Platelet lysates contains several factors that can affect other ion channels or signaling pathways, and thus we assessed the specific role of BK channels on the migration of rMCSs by stimulating the cells with the BK channel agonist NS1619. Similar to the effect observed with 5% PL, BK channel activation with the NS1619 (10 μM) agonist produced a 36% increase in cell migration (1 and 1.36 ± 0.08 for control and NS1619 10 μM, respectively; *n* = 5, *p* < 0.01) (Figures 2A,B). The effect was dose-dependent because cells treated with NS1619 3 μM did not show significant differences in migration versus control cells. Interestingly, rMSCs migration was enhanced by the co-treatment with 5% PL and NS1619 (1.47 ± 0.04 and 1.90 ± 0.07 for 5% PL vs. 5% PL + NS1619 10 μM, respectively; *n* = 5, *p* < 0.001*;* Figures 2A,B) suggesting a synergistic action on BK channels between NS1619 (10 μM) and 5% PL. In addition, BK channel activation or inhibition using NS1619 or IBTX in the presence or absence of 5% PL did not affect cell viability (Figure 2C). These results collectively suggest that PL regulates rMSCs migration through a mechanism that involves the activation of BK channels.

### PL Induces an Increase in rMSC Current Density Without Modifications in BK Channel Expression

**FIGURE 3:**
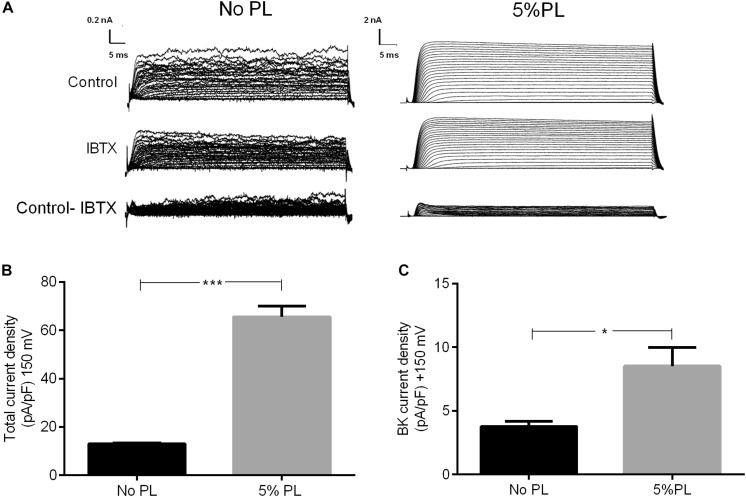
BK channel activity in rMSCs. **(A)** Representative whole-cell currents in response to a pulse protocol (from –100 to +250 mV) before (No PL) and after treatment with 5% PL. Top: Basal currents; middle: currents evoked after IBTX 100 nM treatment; bottom: IBTX-sensitive currents (before minus after addition of IBTX). **(B)** Total current densities in rMSC in the presence and absence of 5% PL. **(C)** BK channel current densities in the presence and absence of 5% PL. Data are shown as mean ± SEM (*n* = 3).

To examine the mechanism by which BK channel regulates rMSCs migration, we used whole-cell patch-clamp recordings to determine whether 5% PL changes BK channel activity. Our results showed that the treatment of rMSCs with 5% PL for 24 h produced an ionic current increase (Figure 3A) including a substantial growth in total current density (13 ± 0.29 and 65.65 ± 4.35 for control and 5% PL, respectively; *n* = 3, *p* < 0.001, Figure 3B). Specifically, PL incubation induced a significant increase in BK currents (3.78 ± 0.42 and 8.52 ± 1.46 for control and 5% PL, respectively, *n* = 3, *p* < 0.01, Figure 3C). These data showed that PL increases cationic outward currents associated with BK channels activity.

**FIGURE 4:**
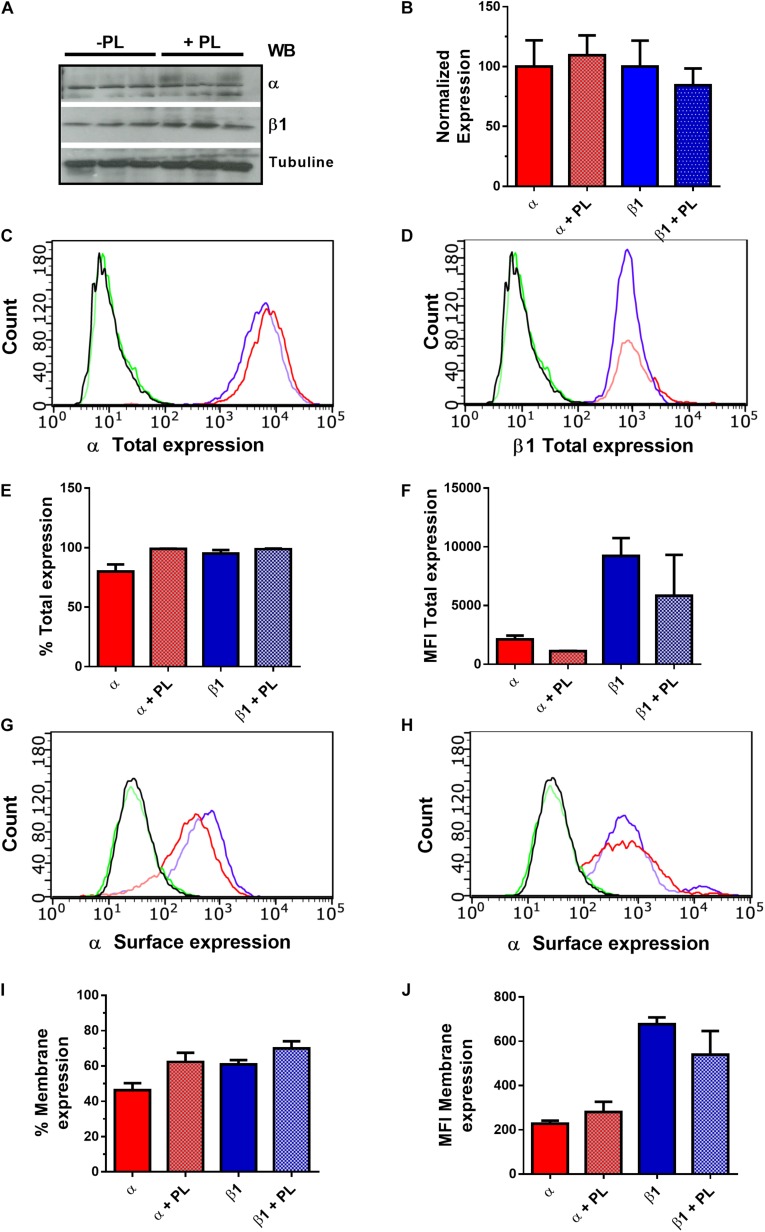
BK channel expression in rMSCs. **(A)** Expression of BK channel α and β1 subunits in rMSCs, analyzed by Western blot in the absence and presence of 5% PL. **(B)** Quantification of BK channel subunits expression by Western blot. Alpha subunit normalization was made using the upper band, that corresponds to the molecular weight of alpha subunit (*n* = 5). Histograms represent total expression of α **(C)** and β1 **(D)** subunits in rMSCs, evaluated by flow cytometry in permeabilized cells. The figure shows data from one representative experiment. Black: cell without staining; Green: Cells with secondary antibody; Red: Control without PL; Blue: Cells treated with 5% PL. **(E)** Quantification levels of total expression measured by flow cytometry (% cells); α: 81.59 ± 5,91, α + PL: 99.12 ± 0.313, β1: 97.60 ± 3,19, β1 + PL: 99.26 ± 0.48. **(F)** BK channel subunits total expression measured by flow cytometry (MFI); α: 2297 ± 350.4, α + PL: 1122 ± 45.3, β1: 9046 ± 1517, β1 + PL: 4964 ± 3472. Histograms represent surface expression of α **(G)** and β1 **(H)** subunits in rMSCs, evaluated by flow cytometry in non-permeabilized cells **(I).** Quantification levels of membrane expression measured by flow cytometry (% cells); α: 44.63 ± 4.10, α + PL: 59.17 ± 5.12, β1: 59.84 ± 2.34, β1 + PL: 67.16 ± 4.21. **(J)** BK channel subunits membrane expression measured by flow cytometry (MFI); α: 219.7 ± 13.96, α + PL: 308.6 ± 47.02, β1: 648.6 ± 30.84, β1 + PL: 559.2 ± 106.8. Error bars: SEM. *n* = 5.

To determine if the PL-dependent increase in BK currents was mediated by changes in BK channel protein expression, the levels of α and β1 subunits in permeabilized cells in the presence or absence of 5% PL were evaluated. The results showed that rMSCs express BK channel α and β1subunits; however, 5% PL (24 h) did not change the total level expression of these subunits (Figures 4A–F). Similarly, the total expression of β2 and β4 subunits in MSCs was not modified by 5% PL (Supplementary Figure S1). Despite the absence of any changes in total expression, PL could also modify the levels of these subunits on the cell surface. To test this possibility, the surface expression of α and β1 subunits was evaluated in non-permeabilized cells. The obtained results showed that 5% PL did not affect the rMSCs surface expression of α and β1 subunits (Figures 4G–J). These results suggest that the PL-induced increase in BK current density was not mediated by modifications in the expression of BK α and β1subunits.

### Effects of PL in Cell Migration Includes Changes in Intracellular Ca^2+^ Concentration [Ca^2+^]i

**FIGURE 5:**
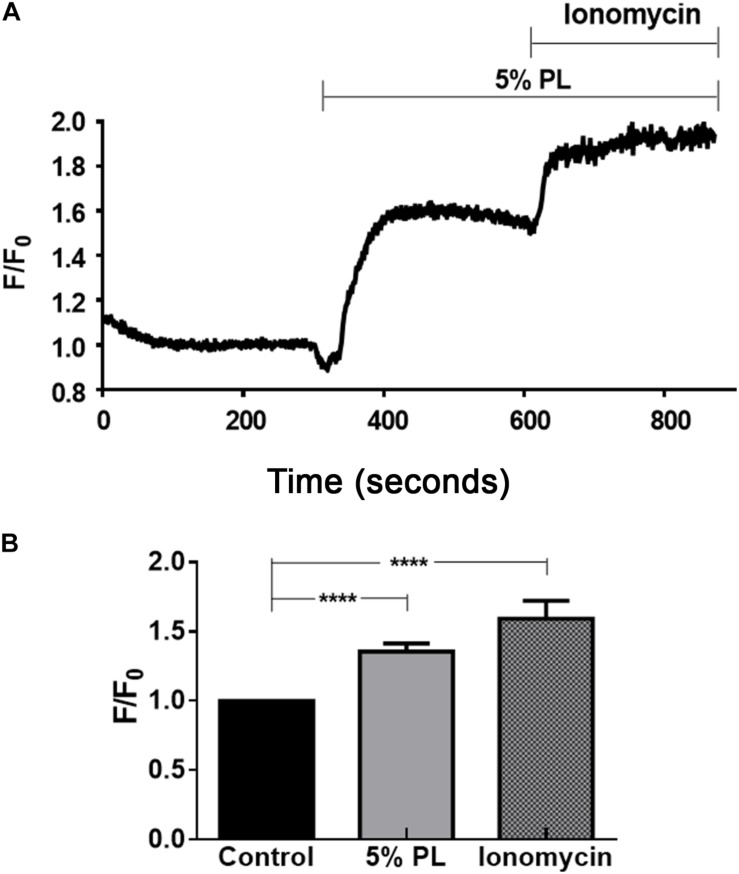
Changes in intracellular calcium concentration induced by 5% PL. **(A)** Representative intracellular calcium imaging traces showing responses to 5% PL in rMSCs. **(B)** Summary histogram of the results obtained in the protocol shown in A. Fluorescence signal was normalized with basal fluorescence PL: 1.26 ± 0.06; Ionomycin: 1.53 ± 0.13. Ionomycin 5 μM was used as a control. *n* = 7–17 cells from 3 to 7 different experiments. Data are shown as mean ± SEM.

Previous studies demonstrated the role of Ca^2+^ in cell migration mechanisms (Dreval et al., 2005; Yang and Huang, 2005). To determine if, PL induces changes in intracellular Ca^2+^ concentration in rMSCs, we evaluated the effect of an acute PL stimulation in the Ca^2+^ levels using Fluo-4AM. The addition of 5% PL increased rMSCs intracellular Ca^2+^ concentration (1 and 1.34 ± 0.06 for control and 5% PL, respectively; *n* = 5, *p* < 0.0001; 1 and 1.59 ± 0.13 for control and ionomycin, respectively; *n* = 5, *P* < 0.0001, Figures 5A,B). This result suggests that PL affects rMSC migration through a mechanism involving changes in intracellular calcium concentration.

## Discussion

In this study, we demonstrated that 5% PL triggers a rise in intracellular Ca^2+^ concentration and increases BK channel activity suggesting that both events are important for the migration of rMSCs. MSCs migration has particular relevance because of its active role in tissue repair. Both endogenous and exogenous MSCs migrate to injured tissue including muscle, lung, brain, bone marrow, and spleen guided by a plethora of signals including cytokines and chemokines (Pisati et al., 2007; Wang et al., 2012; Marquez-curtis et al., 2015).

Previous reports showed that PL has a positive effect on cell migration of monocytes, endothelial cells, fibroblast, keratinocytes, and human MSCs – an improvement in wound healing was observed after PL treatment (Baek et al., 2011; Li and Jiang, 2011; Barsotti et al., 2013; D’Esposito et al., 2015). When we analyzed the effect of PL in rat adipose-derived MSCs migration, we found a 47% increase in migration after treatment with 5% PL. This effect could be associated with PL composition including PDGF, TGF-β1, and vascular endothelial growth factor (VEGF) (Barsotti et al., 2013) because b-FGF, VEGF, hepatocyte growth factor (HGF), insulin-like factor β1 (TGF-β1), and PDGF have critical roles in cell migration and tissue regeneration (Fu et al., 2019).

Ion channel activity is involved in the migration of various cell types through mechanisms associated with changes in channel activity or membrane expression (Steinle et al., 2011; Rosa et al., 2016; Tajhya et al., 2016). Interestingly, growth factors regulate ion channel activation through different mechanism. For example, TGF-β stimulates the activity of epithelial sodium channels (ENaC) expressed in lung (Peters et al., 2014). Similarly, PDGF promotes both activation and membrane expression of acid-sensing ion channel 1a (ASIC1a) in fibrotic mouse liver tissues (Zuo et al., 2019) and increases the activity of Cav1.2 channels in vascular smooth muscle cells (Guo et al., 2018). PDGF and TGF have also been shown to regulate BK channels plasma membrane insertion through the activation of the phosphoinositide 3-kinase/Akt pathway (Holm et al., 1997). In this study, we found that 5% PL treatment increased the BK current density. However, this effect was not associated with changes in total or membrane expression of BK α or β1 subunits suggesting that PL regulates BK current activity through other mechanisms.

BK channels are activated by membrane depolarization and increases in intracellular Ca^2+^ concentration; therefore, they play an essential role in integrating these two cellular processes. We observed that the addition of 5% PL generated a significant increase in intracellular Ca^2+^. Considering previous reports about Ca^2+^ movement in cell migration, it is possible to suggest that Ca^2+^ influx in rMSC stimulated with PL is produced by activation of L-type Ca^2+^ channels (Yang and Huang, 2005). Nevertheless, we cannot discard the Ca^2+^ release from intracellular compartments derived from PL stimulus. The increase in BK channel activity could be associated with a rise in intracellular Ca^2+^, but we do not have sufficient experiments to definitely prove this hypothesis. In addition to voltage and calcium, BK channel activity can be regulated by post-translational modifications such as phosphorylation through protein kinase A (PKA) or G (PKG) (Yang et al., 2010; Kyle and Braun, 2014). Some reports demonstrated that PDFG can induce PKA activation (Eisel et al., 2013) making it feasible that PL-induced post-translational modifications in the BK channel promote an increase in their activity. Future studies are needed to identify the source of cytosolic calcium affected by PL in rMSCs and to unravel the mechanism of BK channel activation by PL.

The 5% PL increased other cationic outward currents in addition to BK channels currents. It is known that MSCs express calcium-activated channels including intermedia and small conductance potassium channels (IK and SK, respectively) (Tao et al., 2007). IK channels are important migration mediators of pancreatic cancer cells and glioblastoma cells where they are overexpressed and mediate K^+^ efflux and changes in cell volume (Bonito et al., 2016; Catacuzzeno and Franciolini, 2018). Moreover, in human monocytes, upregulation and activity of IK channels by intracellular signaling pathways has been linked to migration of these cells (Ma et al., 2018). A role on microglial migration has also been reported for SK channels where these channels participate in Ca^2+^ signaling (Ferreira and Schlichter, 2013). Considering that both IK and SK channels regulate cell migration in other cells (Catacuzzeno et al., 2011; Catacuzzeno and Franciolini, 2018), it is probable that they also have a role in rMSCs migration induced by PL. However, we conclude that the BK channel activity by itself is sufficient to regulate rMSCs migration because inhibition of BK channel is sufficient to abolish the stimulating effect of PL in rMSCs migration.

In summary, the mechanism underlying PL-induced migration involves changes in intracellular Ca^2+^ concentration and increases in BK channel activity. The later event could allow K^+^ efflux, changes in cell volume, and cell movement by retraction of the rear as reported previously in other migrating cells (Schwab, 2001; Schwab et al., 2012). Thus, this study offers progress in determining one of the molecular targets underlying the increase in rMSC migration induced by PL. Finally, we found evidence supporting the use of BK channel modulators in studies that improve migratory properties of rMSCs in regenerative medicine.

### Compliance With Ethical Standards

All experiments were approved by the ethics committee at the Pontificia Universidad Javeriana, Bogotá, Colombia, and conducted following the institutional guidelines for the use of experimental animals. All procedures performed in studies involving human participants were under the ethical standards of the ethics committee at the Pontificia Universidad Javeriana, Bogotá, Colombia, and with the 1964 Helsinki declaration and its later amendments.

## Data Availability Statement

The datasets generated for this study are available on request to the corresponding author.

## Ethics Statement

The animal study was reviewed and approved by the Comité de Investigación y Ética, Pontificia Universidad Javeriana; Acta No. 19 de septiembre 30 de 2010.

## Author Contributions

SE, AG, and SG performed the research and analyzed the data. CSá, CSi and JH performed the research. J-JS analyzed the data and wrote the manuscript. YT designed the experiments, directed the research activities, performed the research, analyzed the data, and wrote the manuscript.

## Conflict of Interest

The authors declare that the research was conducted in the absence of any commercial or financial relationships that could be construed as a potential conflict of interest.

## Supplementary Material

FIGURE S1 BK channel β2 and β4 subunit expression in rMSCs. Quantification levels of total expression measured by flow cytometry (% cells); β2: 15.72 ± 3.57; β2 + PL: 15.62 ± 4.09; β4: 12.95 ± 4.25; β4 + PL: 15.29 ± 4.26.
